# Modulatory Effect of Papaya Extract against Chlorpyrifos-Induced Oxidative Stress, Immune Suppression, Endocrine Disruption, and DNA Damage in Female *Clarias gariepinus*

**DOI:** 10.3390/ijerph19084640

**Published:** 2022-04-12

**Authors:** Abdallah Tageldein Mansour, Heba S. Hamed, Hossam S. El-Beltagi, Walid Fathy Mohamed

**Affiliations:** 1 Animal and Fish Production Department, College of Agriculture and Food Sciences, King Faisal University, P.O. Box 420, Al-Ahsa 31982, Saudi Arabia; 2 Fish and Animal Production Department, Faculty of Agriculture (Saba Basha), Alexandria University, Alexandria 21531, Egypt; 3 Department of Zoology, Faculty of Women for Arts, Science & Education, Ain Shams University, Cairo 11757, Egypt; 4 Agricultural Biotechnology Department, College of Agriculture and Food Sciences, King Faisal University, P.O. Box 420, Al-Ahsa 31982, Saudi Arabia; helbeltagi@kfu.edu.sa; 5 Biochemistry Department, Faculty of Agriculture, Cairo University, Giza 12613, Egypt; 6 Department of Biological and Geological Sciences, Faculty of Education, Ain Shams University, Cairo 11757, Egypt; waledfathy@edu.asu.edu.eg

**Keywords:** *Clarias gariepinus*, chlorpyrifos, *Carica papaya*, oxidative stress, DNA fragmentation, Reproductive hormones, immunity

## Abstract

Chlorpyrifos (CPF) is one of the widely used organophosphorus pesticides in agriculture activities and its presence in the aquatic environment has been broadly recorded. In the present study, we investigated the effect of CPF exposure on oxidative stress, innate immunity, sexual hormones, and DNA integrity of female African catfish, *Clarias gariepinus*, in addition to the potential use of dietary supplementation of papaya, *Carica papaya* (CP), extract against CPF toxicity. Apparent healthy female catfish (300 ± 10 g) were divided into four groups with three replicates each. The first group served as the negative control (fed on a basal diet) and the other groups exposed to CPF (8.75 µg/L) with or without CP extract (250 mg/kg body weight) for six weeks. The results revealed that CPF exposure exhibited marked elevations in stress markers (glucose and cortisol), serum aspartate aminotransferase, alanine aminotransferase activities, testosterone, and luteinizing hormone level. Moreover, CPF increased the percentage of hepatic DNA damage. In addition, catfish exposed to CPF experienced significant decline in serum total protein, albumin, follicles stimulating hormone, estradiol hormone levels, AChE, immunoglobulin, and lysozyme activity. CPF induced significantly oxidative stress in hepatic and renal tissues. The dietary supplementation with CP extract at a level of 250 mg/kg body weight succeeded to alleviate the negative effects of CPF on the physiological, immunological, and antioxidant status of female catfish. In addition, CP extract alleviated the endocrine disruption and hepatic DNA damage and counteracted the subchronic CPF toxicity in female African catfish. Finally, the CP extract may be used as a feed additive in the aquatic diet.

## 1. Introduction

Chlorpyrifos (CPF; O, O-diethyl O-(3,5,6-trichloro-2-pyridyl-phosphorothioate)) is a broad-spectrum organophosphorus pesticide used extensively for the management of domestic and agricultural pests [1]. Its release into the aquatic environment results in toxicity to non-target aquatic species [2]. Therefore, researchers have focused on the toxicity of the CPF on aquatic organisms and their potential adverse effects on the aquatic community [3,4,5]. CPF causes hepatic, neurobehavioral, reproductive, and endocrine dysfunction in fish [6,7,8,9]. It can also lead to oxidative stress [10,11] and genotoxicity in fish [12,13]. In addition, Jiao et al. [14] and Zhang et al. [11] found that common carp exposed to CPF impaired immune functions and caused respiratory disfunction. Xing et al. [3] reported a reduction in acetylcholinesterase and carboxylesterase activities in the brain and muscle of common carp due to CPF exposure.

In the past few years, much more interest has been paid to the use of medicinal plants as feed supplements worldwide to reduce the toxicity induced by different chemicals, heavy metals, drugs, and pesticides [15,16,17,18,19,20,21,22]. *Carica papaya* (family: Caricaceae) is popularly known as pawpaw and papaya [23]. Papaya is cultivated widely all over the world, particularly in tropical countries such as Malaysia, the West Indies, and Africa [24,25]. *C. papaya* is known to have antioxidant and immune-modulating activities and is traditionally utilized for medicinal purposes [26]. The papaya fruit has a delicious taste and is a good source of carotene, vitamin C, vitamin B, flavonoids, folate, pantothenic acid, potassium, and magnesium [27,28]. Additionally, Li et al. [29] reported that the active constituents of papaya including chymopapain and papain are utilized to treat joint pain and digestive issues. Extracts of ripe fruits are additionally utilized for an assortment of medicinal purposes, for example, medications for treating ringworm, intestinal sickness, and hypertension. Furthermore, papaya includes a range of phytochemicals such as natural phenols [30] and polyphenols which prevent biomolecules oxidation through their free radical scavenging capabilities [31] or by increasing the activity of endogenous antioxidant enzymes [32].

African catfish, *Clarias gariepinus*, is a cosmopolitan ‘big’ Clarias species with wide distribution in Africa and several temperate regions around the world. Its high demand in aquaculture derives not only in growing to a big size but also from being hardy to withstand stress [33]. Thus, *C. gariepinus* is considered a suitable model for toxicological studies [34,35,36,37]. To the best of our knowledge, most of the toxicity studies have used male catfish as experimental models except few studies which revealed degenerative changes in the ovary including ooplasm vacuolization, follicular lining damage, and deshaped oocytes [38]. In addition, lead and mercury exposure induced development impairments in the ovaries and degenerative vitologenic oocytes [39] and significantly increased the percentage of germinal vesicles breakdown [40]. Therefore, the present study was designed to evaluate the toxicity of CPF on sexual hormones level, redux status, physiological and immunological status, and DNA integrity of female catfish, *C. gariepinus*. In addition, determining the palliative effect of *C. papaya* extract against the toxicity of CPF was investigated.

## 2. Materials and Methods

### 2.1. Plant Authentication, Aqueous Extraction, and Bioactive Components of the Extract

Mature fresh papaya, *C. papaya* (CP), fruit was obtained during the winter of 2020 from the Ministry of Agriculture and Land Reclamation, Cairo, Egypt. The fruits were identified in the Horticulture Department, Faculty of Agriculture, Ain Shams University, and the voucher was kept in the herbarium of the same department. The fruit was peeled and the cream-hued seeds were discarded. The aqueous extract was obtained as follows: a total of 1000 g of the fruit was mixed well with 1000 mL of physiological saline (sodium chloride 0.9%) and incubated at room temperature for 72 h [41,42]. The extract was then sieved into a clean container and stored in a refrigerator until use. The used concentration of CP aqueous extract was 250 mg/kg body weight as dietary supplementation according to Abouzed et al. [43]. The identification of bioactive components of the CP extract was conducted by gas chromatography-mass spectrometry (GC-MS). The most abundant identified phenolic compounds in CP extract were 16.0% caffeic acid and 13.0% flavonoid quercetin-3-O-rutinoside. In addition, several phytochemicals were recorded such as 1.6% ferulic acid, 5.0% rutin, 0.8% gallic, and 0.6% protocatechuic acids conjugates. The other phytocomponents found in CP in terms of their relative abundance were tetradecanoic acid, octadecanoic acid, hexadecanoic acid, and methyl ester.

### 2.2. Fish Maintenance

A total of 350 aberrant healthy female catfish, *C. gariepinus* (average weight 300 ± 10 g, length 40 ± 3 cm), were obtained from Abassa fish farm, Sharqia governorate, Egypt. Fish were transferred to the laboratory in 100 L well-aerated fiberglass tanks. Fish were treated with (0.5% *w*/*v*) potassium permanganate solution for 1 min. to remove dermal adherents. The fish were acclimatized for 2 weeks in identical glass aquaria measuring 80 × 60 × 40 cm having 85 L of dechlorinated aerated tap water under laboratory conditions. The daily water exchange rate was 30%. The water quality parameters were as follows: pH was 7.5 ± 0.3, temperature was 28 ± 1 °C, dissolved oxygen was 6.5 ± 0.4 mg/L, alkalinity was 122 mg/L, and hardness was 152 mg/L CaCO_3_. Fish were fed on a commercial diet (Aller-Aqua, Giza Governorate, Egypt) at a rate of 3% of body weight per day. The proximate chemical composition of the used diet was 32.00% crude protein, 8.00% crude lipid, 4.5% fiber, and 8.5% ash.

All procedures and handling methods used in the current study had been accepted by the Research Ethical Committee (R.E.C.) of Faculty of Women for Arts, Science & Education, Ain shams University, Cairo, Egypt.

### 2.3. Experimental Design

#### 2.3.1. Determination 96 h LC_50_ of Chloropyrofis

The half-lethal concentration (LC_50_) of Chloropyrofis (CPF; National Company for Fertilizers and Chemicals, Cairo, Egypt) was determined with the definitive test by the static renewal bioassay method of Litchfield and Wilcoxon [44]. Briefly, five groups each of eight fish were exposed to various concentrations of CPF (0, 25, 50, 100, 200, or 400 µg/L.). The half-lethal concentration (LC_50_) of chlorpyrifos was determined according to the following formula:

LC_50_ = the highest concentration − ∑a × b/n (1)

where a: constant factor of difference between groups; b: mean value of dead fish between every two successive groups; and n: number of fish in each group.

#### 2.3.2. Sublethal Exposure

After the acclimatization period, catfish were distributed into four groups in triplicates containing 12 fish and exposed to the following treatments for six weeks: Fish in the 1st group was fed the basal diet and reserved as the control group. Fish in 2nd group was exposed to 10% of the LC_50_ of CPF (8.75 µg/L). Fish in 3rd group was dietary administered CP extract (250 mg/kg body weight). Fish in 4th group was exposed to CPF (8.75 µg/L) and administered CP extract (250 mg/kg body weight). In the CPF exposed groups, with each water exchange, the new water was dosed with the same CPF concentration to maintain the same exposure level throughout the experiment.

#### 2.3.3. Blood Sampling

Six fish from each treatment were collected at the end of the experiment and anaesthetized with 0.02% benzocaine solution. Blood samples were taken from the caudal arteries and allowed to clot at room temperature in clean dry centrifuge tubes before being centrifuged at 800× *g* for 15 min at 4 °C to determine biochemical and immunologic characteristics.

#### 2.3.4. Biochemical Assays

All biochemical assays were conducted using commercial kits (Bio-Diagnostic Co., Cairo, Egypt). Serum glucose and cortisol levels were assessed using the methods described by Foster and Dunn [45], respectively. Serum aspartate aminotransferase (AST) and alanine aminotransferase (ALT) were detected by [46]. Serum total protein and albumin were measured using the methods described by Henry [47] and Doumas et al. [48], respectively. Serum globulin was determined by subtracting the albumin value from the total protein value of the same sample. The AChE activity was determined according to [49].

#### 2.3.5. Determination of Tissue Lipid Peroxidation and Antioxidant Enzyme Activities

Samples from hepatopancreas and renal tissues were homogenized in cold phosphate buffer saline (0.1 M, pH 7.4) using a Potter-Elvejhem glass and Teflon homogenizer. The homogenate was centrifuged at 1600× *g* at 4 °C for 10 min. Supernatants were stored at −20 °C until analysis. Lipid peroxidation (LPO) levels were determined according to Uchiyama and Mihara [50]. Superoxide dismutase (SOD) activity was determined using the method described by Nishikimi et al. [51]. Catalase (CAT) was measured according to the method of Aebi [52]. Total antioxidant capacity (TAC) and reduced glutathione (GSH) levels were estimated using the method of Koracevic et al. [53] and Beutler [54], respectively, using commercial kit (Gamma Trade Co., Cairo, Egypt).

#### 2.3.6. Immunological Assays

According to Ellis (1990), serum lysozyme (LYZ) was measured using a turbidimetric technique using Micrococcus luteus as the target in phosphate buffer (pH = 6.2). (1990). According to Secombes [55], the respiratory burst (RB) activity of the whole blood sample was assessed using Nitroblue Tetrazolium dye. The IgM was precipitated in polyethylene glycol, the initial and final total proteins were subtracted, and immunoglobulin (IgM) was quantified [56].

#### 2.3.7. Reproductive Hormones Examination

Serum 17-β Estradiol (E2) and follicle-stimulating hormone (FSH) levels were assessed by ELISA according to Abraham [57] and Knobil [58], respectively. Luteinizing hormone (LH) and testosterone levels were detected according to the method of Tietz et al. [59], respectively, using commercial kit (Gamma Trade Co., Cairo, Egypt).

#### 2.3.8. Hepatic DNA Fragmentation Measurement

The DNA fragmentation of hepatic specimens of female catfish was determined according to the method described by Kurita-Ochiai et al. [60] using a spectrophotometer (Micro lab 200 Vital Scientific Dieren, The Netherlands) at 575 or 600 nm against reagent blank. The percentage of fragmented DNA was assessed by the following formula:

% of fragmented DNA = fragmented DNA/(fragmented DNA + intact DNA) × 100(2)

### 2.4. Statistical Analyses

The results were presented as means ± standard error. Data were statistically analyzed using analysis of variance, one-way ANOVA, to evaluate effects of CPF toxicity and dietary PC extract. Differences among means were tested at the 5% probability level using Duncan’s multi-comparison test. All the statistical analyses were done using SPSS (version 20; SPSS, Richmond, VA, USA).

## 3. Results

### 3.1. Determination of the 96 h LC_50_ of Chlorpyrifos

The 96 h LC_50_ value of CPF was determined as 87.5 µg/L (Table 1) and a sublethal concentration of CPF equal to 10% of the LC_50_ (8.75 µg CPF/L) was used in the current investigation as chronic stressors.

### 3.2. Biochemical Analysis

Female African catfish exposed to a sublethal level of CPF showed marked elevations in levels of serum liver enzyme activities (AST, ALT), glucose, and cortisol levels compared to the control fish (Table 2). In comparison to the control tilapia, there was a considerable decrease in serum AchE, total protein, albumin, and globulin levels (Table 2). On the contrary, CP extract (250 mg/kg bw) into the diet along with CPF-exposed catfish normalized levels of all the tested parameters to near the levels of the control group (Table 2). Fish of the control group (1st group) and fish treated with CP extract (2nd group) had nearly the same values of the tested biochemical parameters.

### 3.3. Lipid Peroxidation and Antioxidant Enzyme Activities

**Figure 1:**
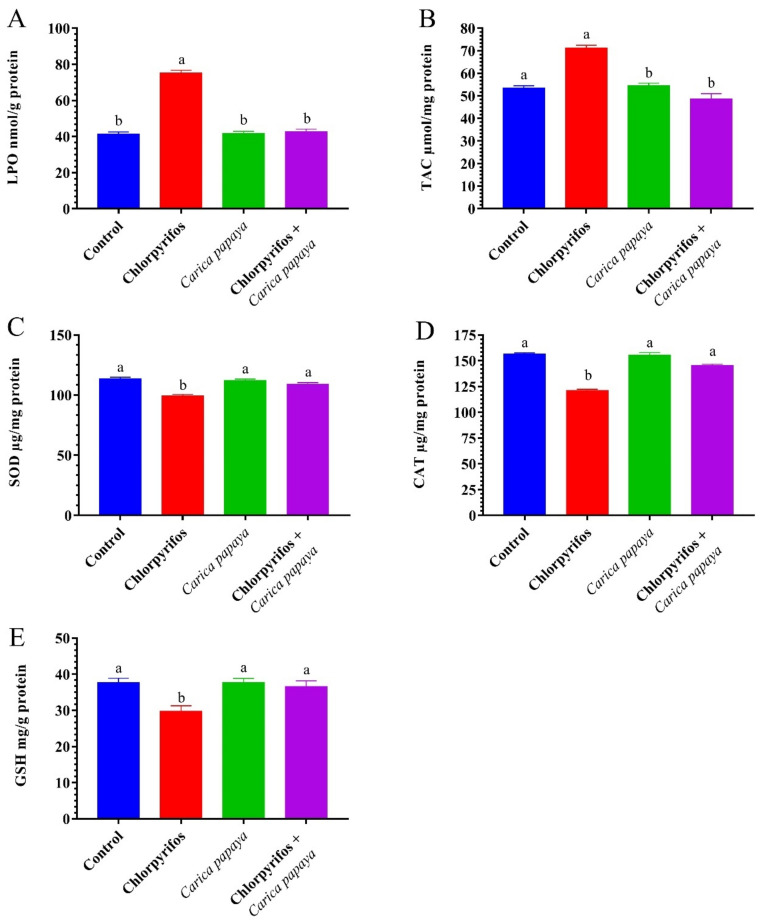
Lipid peroxidation (**A**), total antioxidant capacity (**B**), superoxide dismutase (**C**), total antioxidant capacity (**D**), catalase (**D**), and reduced glutathione (**E**) of hepatopancreas tissue in female African catfish, *Clarias gariepinus*, exposed to chlorpyrifos (8.75 µg/L.) and co-administrated with papaya, *Carica papaya*, aqueous extract (250 mg/kg body weight) for 6 weeks. The column bearing with different letters are significantly different (*p* < 0.05).

**Figure 2:**
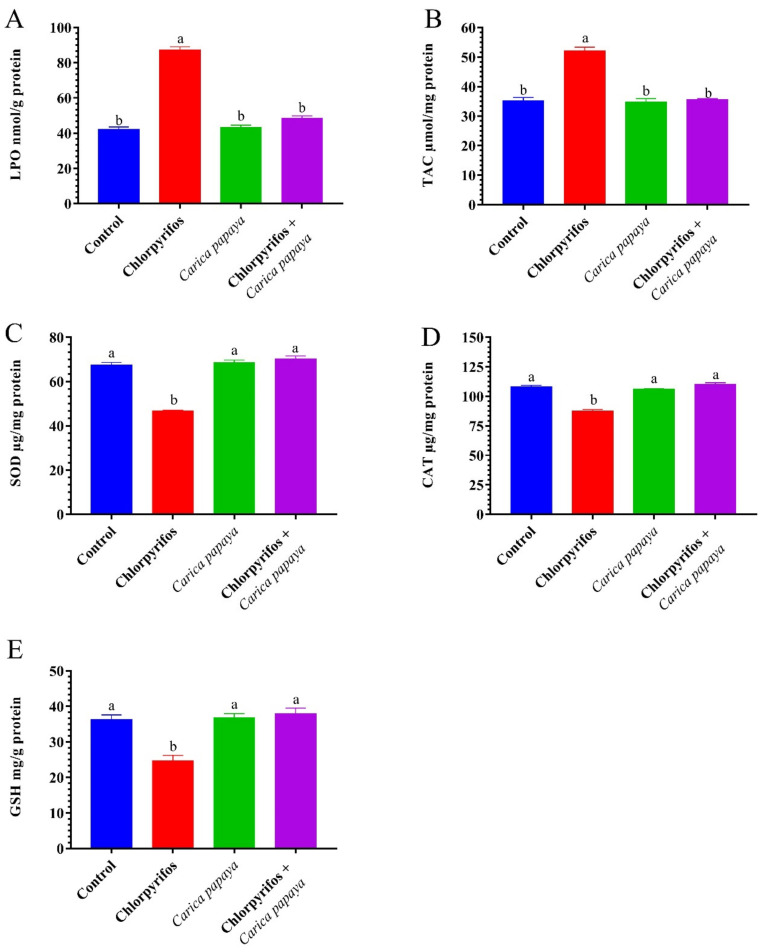
Lipid peroxidation (**A**), total antioxidant capacity (**B**), superoxide dismutase (**C**), total antioxidant capacity (**D**), catalase (**D**), and reduced glutathione (**E**) of renal tissue in female African catfish, *Clarias gariepinus*, exposed to chlorpyrifos (8.75 µg/L) and co-administrated with papaya, *Carica papaya*, aqueous extract (250 mg/kg body weight) for 6 weeks. The column bearing with different letters are significantly different (*p* < 0.05).

The exposure of female catfish to CPF caused a marked (*p* < 0.05) increase in hepatorenal LPO and TAC levels as presented in Figure 1 and Figure 2. The CAT, SOD, and GSH activities significantly decreased in the liver and kidney tissues compared to the control group (Figure 1 and Figure 2). The administration with CP extract 250 mg/kg bw of showed no significant difference in hepatorenal antioxidant enzyme activities compared to the control group. Treatment of CPF-exposed fish with CP extract resulted in a significant decrease in LPO and TAC levels and a marked an improvement in hepatorenal oxidative stress enzymes.

### 3.4. Innate Immune Response

**Figure 3:**
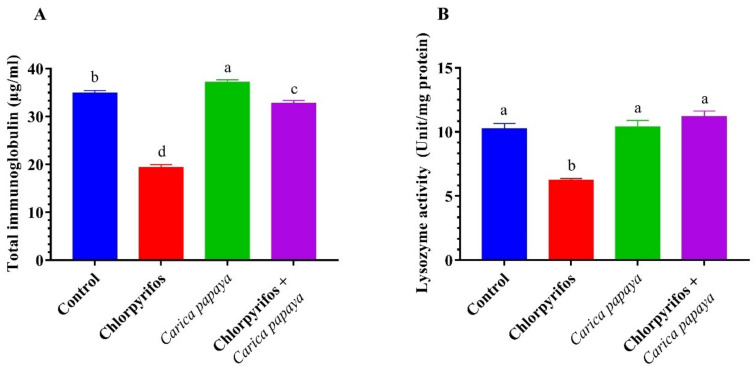
Total immunoglobulin (**A**) and lysozyme (**B**) of female African catfish, *Clarias gariepinus*, exposed to chlorpyrifos (8.75 µg/L.) and co-administrated with papaya, *Carica papaya* aqueous extract (250 mg/kg body weight) for 6 weeks. The column bearing with different letters are significantly different (*p* < 0.05).

Catfish exposed to (8.75 µg/L.) CPF for 6 weeks exhibited a significant reduction in the lysozyme activity and immunoglobulins levels (Figure 3). The combined treatment of CPF and CP extract resulted in a marked enhancement in the lysozyme activity and immunoglobulins levels.

### 3.5. Reproductive Hormones Analysis

The concentrations of 17-β E_2_ and FSH in female catfish were significantly reduced during CPF exposure (Table 3). On the other hand, levels of LH and testosterone were markedly elevated (*p* < 0.05) in the group exposed to CPF (8.75 µg/L.) for 6 weeks (Table 3). Co-administration of CP extract (250 mg/kg bw) with CPF exposure caused a significant increase in serum FSH and 17-β E_2_ levels and restored the concentrations of LH and testosterone to the normal values in comparison with CPF-exposed fish (Table 3).

### 3.6. Liver DNA Fragmentation

**Figure 4:**
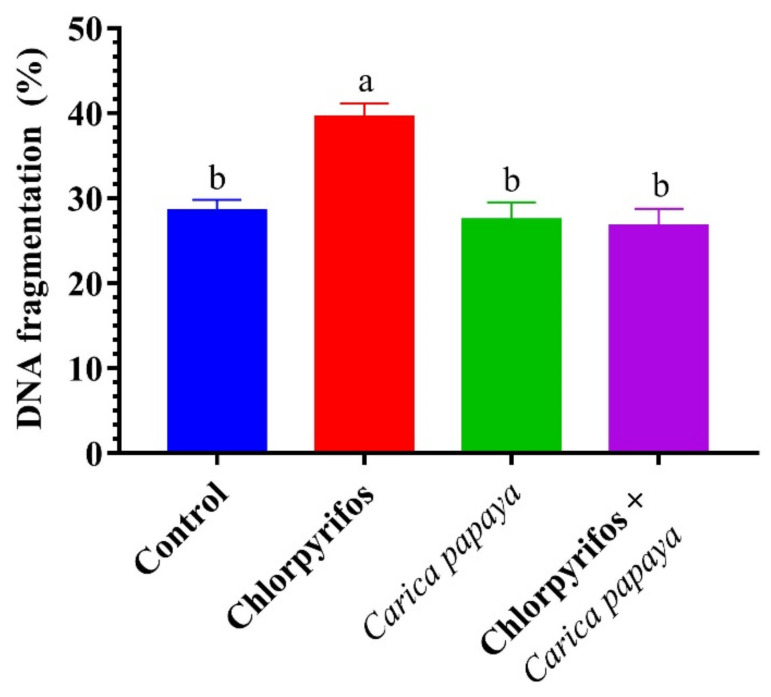
DNA fragmentation (%) of female African catfish, *Clarias gariepinus*, exposed to chlorpyrifos (8.75 µg/L.) and co-administrated with papaya, *Carica papaya* aqueous extract (250 mg/kg body weight) for 6 weeks. The column bearing with different letters are significantly different (*p* < 0.05).

CPF-exposed fish showed a significant (*p* < 0.05) increase in the percentage of hepatic DNA fragmentation when compared to the control catfish, as seen in Figure 4. In contrast, combined treatment with CPF and CP extract showed a significant decrement in the percentage of hepatic DNA fragmentation compared to the CPF-intoxicated group (Figure 4).

## 4. Discussion

The 96 h LC_50_ value of CPF in female African catfish was found to be 87.5 µg/L. In similar studies, the 96 h LC_50_ of CPF in *O. mossambicus* with a mean body weight 3.0 g was 25.97 µg/L [61]. Meanwhile, the 96 h LC_50_ values of technical grade (94% a.i.) and commercial CPF (20% EC) on Nile tilapia with average body weight 2.64 ± 0.16 g were 109 µg/L and 47 µg/L, respectively [5]. They attributed the difference in CPF toxicity on fish to the type of CPF where they suggested that the technical grade (active ingredient) of CPF was more toxic to fish than its emulsified concentration. In addition, Paracampo et al. [62] found that the 96 h LC_50_ of CPF was 105 µg/L for *Cnesterodon decemmaculatus* with a maximum length of 25 and 45 mm for males and females, respectively. The 96 h LC_50_ value of CPF for *Cyprinus carpio* with mean body weight (47.51 ± 3.24 g.) was 0.160 mg/L [63]. Meanwhile, Zhang et al. [11] found that the 96 h LC_50_ of CPF in *C. carpio* (average body weight of 15.00 ± 5.0 g) was 149.0 μg/L. The sensitivity of fish to CPF toxicity could be related to many different reasons such as age, fish species, exposure period, fish size, and ecological circumstances.

In the present study, serum AST and ALT exhibited high values in CPF-exposed catfish without feeding on CP extract. The results showed that the hepatic tissue of catfish was severely impaired by CPF exposure while CP extract represented a functional remedy for fish. In accordance with the present findings, African catfish exposed to CPF or other insecticides had higher blood levels of AST and ALT [34,64,65], although other research found a significant drop in liver enzyme levels when feed additives were added to fish diets [17,64]. In addition, the CP extract had more hepatoprotection against CCl4 toxicity than vitamin E. Hence, the results indicated that CP extract possesses hepatoprotective properties [66].

In the same way, glucose and cortisol displayed high levels in fish exposed to CPF [36,67]. This hyperglycemia seems to be a sign of stress in fish exposed to toxicity and is also associated with an elevation in cortisol [17,68] to cope with the toxicity effects as an attempt to cover the energy demands [69]. The results also showed a marked reduction in AChE of catfish exposed to CPF for 6 weeks. Salbego et al. [70] described the inhibition of AChE to the oxidative stress generated by pesticide exposure which provides a convenient non-destructive means of monitoring exposure to pesticides [71]. Many researchers have focused on the ability of CPF to cause hyperglycemia where exposure to CPF decreased serum insulin levels and increased serum glucose levels (hyperglycemia) [72,73,74]. Degeneration and necrosis of glandular acini and beta cells of the pancreas with a proliferation of interlobular ducts in broilers after exposure to CPF supported the alterations in the level of insulin and glucose [75]. Pancreatic beta cells contain muscarinic receptors which play a vital role in insulin production and secretion [76]. By inhibiting AChE activity, CPF increases the accumulation of acetylcholine leading to more stimulation of its receptors and subsequently down-regulates these receptors [77], causing a reduction in insulin synthesis [78]. Furthermore, prolonged stimulation by AChE may reduce beta cell sensitivity to glucose [79]. Hence, inhibition of AChE activity plays a role to some extent in CPF-induced hyperglycemia [80]. This is consistent with the findings of Narra et al. [1] and Hamed and El-Sayed [15] in which the crab *Barytelphusa guerini* was treated with CPF and *Oreochromis niloticus* exposed to pendimethalin. In the present study, the feeding of CPF-exposed catfish on the supplemented diet with CP extract reduced glucose and cortisol levels followed by an increment in AChE activity. These findings could be attributed to the bioactive compounds of CP which stimulates glucose uptake exhibiting hypoglycemia. Similar results were documented in Nile tilapia fed diets containing green tea leaves [20] and *Moringa oleifera* leaves [15] and CPF also diminished the levels of total protein, albumin, and globulin in catfish. The vascular leakage and high proteolysis rate are to account for the lower total protein level [81,82,83], while the decreased albumin level can be attributed to the high rate of renal excretion and failure of protein synthesis due to liver malfunction in the fish exposed to CPF [84,85]. Meanwhile, feeding with CP extract restored the levels of total protein, albumin, and globulin in female catfish exposed to CPF for six weeks. The enhanced serum total protein and its derivatives in CPF-exposed catfish co-administrated with dietary CP extract suggest immune-stimulatory effects of CP in African catfish. In addition, the co-treatment of cypermethrin-intoxicated Nile tilapia with guava leaves extract enhanced serum total protein, albumin, and globulin levels [17].

Environmental contaminants or their metabolites produce oxidative damage in fish [18,69,86,87,88]. In the present study, the antioxidant profile in catfish exposed to CPF exhibited a marked rise in hepatorenal LPO and TAC and declines in the levels of TAC, CAT, SOD, and GSH. The LPO or oxidation of polyunsaturated fatty acids has been observed and used to monitor the effect of pollutants [89,90,91,92]. In the present study, the LPO level increased significantly in the hepatic and renal tissues of female *C. gariepinus* after exposure to CPF for six weeks. Increased LPO levels could be attributed to the inequality state between the production and removal of reactive oxygen species (ROSs) in the cell due to oxidative stress which allows ROSs to attack the lipids, proteins, and DNA of the cell leading to mitochondrial dysfunction and DNA fragmentation which finally causes cell death [93]. CP extract inclusion in catfish feeds markedly decreased the LPO levels due to its constituents of antioxidant phytochemicals such as lycopene, vitamin C, vitamin E, beta-carotene, quercetin, flavonoids, and phenolic compounds which scavenge free radicals generated after CPF exposure [26,94,95].

In all living beings, ROSs are transformed into bland metabolites by antioxidant enzymes (SOD and CAT), which may decrease or increase under chemical exposure [96]. In the present study, the SOD and CAT activities decreased in the hepatic and renal tissues of catfish exposed to 8.75 µg CPF/L. The inhibitory effect may be a result of the overproduction of ROSs induced by CPF exposure, whereas the SOD-CAT system is considered the first line of defense against oxidative damage. The SOD catalyzes the conversion of superoxide anions (O^2−^) into H_2_O_2_ and CAT converts H_2_O_2_ into H_2_O and O_2_ [17,97]. In a similar study, Kadry et al. [98] reported that antioxidant enzyme activities were reduced in the liver of female *C. gariepinus* exposed to atrazine for six weeks. In addition, Abdel-Daim et al. [99] found a marked decline in CAT and SOD activities of tilapia fish exposed to CPF. Dietary supplementation of CP extract in the catfish diet resulted in enhancement of the action of the antioxidant enzymes CAT and SOD activities and TAC level through the removal of excess ROSs to combat oxidative stress. These positive effects could be due to the PC extract content of antioxidant phytochemicals.

The observed reduction in GSH values in liver and kidney tissues of CPF exposed catfish is considered a primary concern of CPF-induced oxidative stress. The reduction in GSH could be due to its depletion to oppose oxidative destruction in response to pollutant exposure [36]. In addition, Shaban et al. [100] explained the decrease in GSH level due to the high demand and consumption of the tripeptide for lipid hydroperoxide metabolism. Similar studies supported this result [101]. The co-administration of CP aqueous extract restored the GSH levels in hepatorenal tissues of catfish after CPF exposure compared with the control fish, where CP acted as an antioxidant and subsequently reduced the consumption of GSH.

In the present study, the total immunoglobulin and lysozyme activity experienced low values in CPF-intoxicated catfish suggesting an immune suppression effect of CPF. Several insecticides could induce immune suppression effects in the fish by altering the transcription of important mediators of the fish’s immune system [17,102]. Feeding catfish a CP-enriched diet during exposure to CPF for six weeks substantially restored the innate immunity parameters, indicating an enhancement of the non-specific innate immunity defense in fish. The results could be attributed to the phytochemical compounds of CP aqueous extract such as lycopene, vitamin C, vitamin E, beta-carotene, and quercetin [26,94,95] as well as the proteolytic enzymes in CP aqueous extract (papain and chymopapain) which could enhance feed utilization and improve the general animal performance [103]. Accordingly, the CP extract revealed a potential immune stimulant property on fish and could alleviate the immunotoxicity effects of CPF. In similar study, the dietary guava leaf powder exhibited immune-stimulatory effects against cypermethrin toxicity in Nile tilapia [17]. In addition, [104] found that dietary pomegranate peel powder ameliorated the immune-toxicological effects of silver nanoparticles on Nile tilapia.

Endocrine disruption is another adverse effect of environmental pollutants. The pituitary hormones (LH and FSH) play an important role in the reproductive process [105,106]. The results of the current work showed significant reductions in concentrations of FSH and 17- β E_2_ hormones and noticeable elevations in LH and testosterone levels after exposure of female catfish to CPF for 6 weeks. The increase in testosterone in female catfish after exposure to CPF could be attributed to increasing the activity of 17 alpha-hydroxylase enzymes involved in testosterone synthesis. Similar studies [71] recorded that testosterone levels increased in female catfish exposed to bisphenol-A. Additionally, the LH hormone of juvenile African catfish was significantly increased after exposure to nonyl phenol for 2 weeks [107]. Our results are also in accordance with da Silva et al. [108] who found that estradiol concentrations decreased in rats intoxicated with bisphenol-S during pregnancy and lactation stages. The reduction in the estradiol level could be attributed to the hypothalamus-pituitary-ovarian axis which was damaged by CPF. The hormonal changes in gonads of CPF-intoxicated female catfish reflect their sensitivity to CPF, suggesting that CPF has androgenic effects. Co-administration with CP aqueous extract (250 mg/kg bw) caused significant improvements in reproductive hormone concentrations of CPF-exposed fish. This improvement may be because of flavonoids, polyphenols, and antioxidant components in the CP aqueous extract [109]. Likewise, the quercetin that was reported in CP extract protected against deltamethrin- and cypermethrin-induced reproductive system toxicity and oxidative damage in rats [95]. This demonstrated that CP aqueous extract has a hormonal recovery effect against CPF.

The current investigation exhibited that exposure of catfish to CPF at a level of 8.75 µg/L resulted in a marked increase in the percentage of liver DNA fragmentation. CPF has the potential to generate mutagenic impacts by generating oxidative DNA damage [25,109]. These genotoxic effects of CPF could be attributed to the generation of free radicals and the imbalance in calcium homeostasis due to the damage in the endoplasmic reticulum membrane [64]. Dietary treatment of female catfish with CP extract at a level of 250 mg/kg body weight reduced the percentage in DNA damage of hepatic tissues after exposure to CPF via oxidation by H_2_O_2_. This study suggests that feeding catfish on CP extract had a powerful antioxidant effect that succeed to reduce the adverse effects of free radicals and inhibit the oxidation of molecules such as lipids and DNA. Hence, CP induced a significant depletion in the elevation level of DNA fragmentation in liver tissues of CPF-intoxicated catfish.

## 5. Conclusions

According to the present findings, it could be concluded that the exposure of female African catfish *Clarias gariepinus* to chlorpyrifos (CPF) caused variations in serum biochemical, immunological assays, and oxidative damage in different tissues. These parameters could also be considered good indicators for monitoring pollution in the aquatic ecosystem including fish. On the other hand, dietary supplementation with papaya *Carica papaya* (CP) extract could improve the physiological and immunological status and alleviated hormonal disruption of catfish exposed to with CPF. Consequently, feeding fish with a CP-extract-enriched diet could minimized the negative impacts of CPF toxicity.

## Figures and Tables

**Table 1:** Half-lethal concentration (LC_50_) of Chlorpyrifos (CPF) in female African catfish, *Clarias gariepinus*.

CPF Concentration (µg/L)	No. of Fish/Concentration	No. of Alive Fish	No. of Dead Fish	a	b	a × b
0.00	8	8	0	0.0	0.0	0.00
25.00	8	6	2	25	1.00	25
50.00	8	4	4	25	3.00	75
100.00	8	2	6	50	5.00	250
200.00	8	1	7	100	6.50	650
400.00	8	0	8	200	7.50	1500
				∑ a × b =		2500

Half-lethal concentration (LC_50_) of chlorpyrifos = the highest concentration − ∑a × b/n; a: constant factor of difference between groups; b: mean value of dead fish between every two successive groups; and n: number of fish in each group.

**Table 2:** Blood biochemical parameters in female African catfish exposed to chlorpyrifos (8.75 µg/L.) and/or co-administrated with papaya, *Carica papaya*, aqueous extract (250 mg/kg body weight) for 6 weeks.

Items	Control	Chlorpyrifos	Papaya	Chlorpyrifos + Papaya
Aspartate aminotransferase (µ/L)	48.15 ± 0.47 ^b^	62.14 ± 0.51 ^a^	46.21 ± 0.84 ^b^	48.29 ± 0.13 ^b^
Alanine aminotransferase (µ/L)	18.05 ±0.37 ^a^	10.21 ± 0.04 ^b^	17.92 ± 0.34 ^a^	17.41 ± 0.087 ^a^
Glucose (mg/dL)	86.87 ± 0.34 ^b^	120.31 ± 1.31 ^a^	86.57 ± 0.41 ^b^	88.05 ± 0.36 ^b^
Cortisol (µg/dL)	11.66 ± 0.28 ^b^	21.21 ± 0.09 ^a^	11.57 ± 0.19 ^c^	13.51 ± 0.16 ^b^
Acetylcholinestrase (µ/L)	545.23 ± 1.16 ^a^	381.27 ± 2.27 ^c^	545.17 ± 2.18 ^a^	550.35 ± 1.35 ^b^
Total proteins (g/dL)	6.53 ± 0.12 ^a^	3.14 ± 0.26 ^b^	6.64 ± 0.17 ^a^	6.35 ± 0.01 ^a^
Albumin (g/dL)	3.87 ± 0.12 ^a^	1.95 ± 0.21 ^b^	3.75 ± 0.11 ^a^	3.89 ± 0.09 ^a^
Globulin (g/dL)	2.66 ± 0.11 ^a^	1.19 ± 0.01 ^b^	2.89 ± 0.31 ^a^	2.46 ± 0.14 ^a^

Means with different superscript letters in the same row for each parameter are significantly different (*p* < 0.05).

**Table 3:** Reproductive hormones in female African catfish, *Clarias gariepinus*, exposed to chlorpyrifos (8.75 µg/L.) and/or co-administrated with papaya, *Carica papaya*, aqueous extract (250 mg/kg body weight) for 6 weeks.

Items	Control	Chlorpyrifos	Papaya	Chlorpyrifos + Papaya
Follicle-stimulating hormone (µ/L)	0.68 ± 0.05 ^a^	0.26 ± 0.06 ^c^	0.72 ± 0.04 ^a^	0.67 ± 0.03 ^b^
Luteinizing hormone (µ/L)	0.33 ± 0.01 ^b^	0.78 ± 0.09 ^a^	0.31 ± 0.04 ^b^	0.32 ± 0.05 ^b^
Estradiol (17-β E2; g/mL)	349.31 ± 0.41 ^a^	253.51 ± 0.15 ^c^	350.21 ± 0.23 ^a^	339.24 ± 0.13 ^b^
Testosterone (g/mL)	54.15 ± 0.34 ^b^	80.52 ± 0.25 ^a^	55.20 ± 0.41 ^b^	50.34 ± 0.31 ^c^

Means with different superscript letters in the same column for each parameter are significantly different (*p* < 0.05).

## Data Availability

The data that support the findings of this study are available from the authors upon reasonable request.
